# Predicting Physical Activity in Chinese Pregnant Women Using Multi-Theory Model: A Cross-Sectional Study

**DOI:** 10.3390/ijerph192013383

**Published:** 2022-10-17

**Authors:** Wei Zhang, Ying Jin, Ningning Liu, Zhenzhen Xiang, Xiaojuan Wang, Ping Xu, Pingping Guo, Minna Mao, Suwen Feng

**Affiliations:** 1 Women’s Hospital, School of Medicine Zhejiang University, Hangzhou 310006, China; 2 Faculty of Nursing, School of Medicine Zhejiang University, Hangzhou 310052, China

**Keywords:** physical activity, pregnant women, multi-theory model, exercise

## Abstract

Background: Physical activity (PA) brings many benefits to pregnant women and fetuses; however, the majority of pregnant women do not participate actively in PA during pregnancy. Objectives: This study aimed to: (1) assess the utility of Multi-Theory Model (MTM) to explain the intentions of PA behavior in Chinese pregnant women; (2) analyze the predictors in initiating and maintaining PA behavior based on MTM. Methods: A cross-sectional study including pregnant women was conducted from March to June 2022 at a university hospital in Hangzhou, Zhejiang Province, China. Participants completed measures that included a self-developed demographic questionnaire and a 29-item MTM questionnaire. Descriptive statistics and stepwise multiple regression were used to analyze the data. The reliability was assessed by Cronbach’s alpha and test-retest stability. The construct validity was evaluated by using exploratory factor (EFA) analysis and confirmatory factor analysis (CFA). Results: A total of 450 pregnant women participated in this study. The score of the magnitude of intention to initiate and maintain PA behavior during pregnancy was 2.30 (1.08) and 2.24 (1.09). The overall Cronbach’s alpha value was 0.857. A four-factor structure for initiation model and a three-factor structure for maintenance model were determined. Results of the CFA confirmed construct validity of subscales (initiation model: χ^2^ = 206.123, df = 140, *p* < 0.001, χ^2^/df = 1.472, RMSEA = 0.046, SRMR = 0.0432, GFI = 0.913, CFI = 0.982; maintenance model: χ^2^ = 49.742, df = 29, *p* < 0.001, χ^2^/df = 1.715, RMSEA = 0.057, SRMR = 0.0432, GFI = 0.958, CFI 0.985). The result of regression indicated that participatory dialogue (β = 0.030; *p* = 0.002), behavioral confidence (β = 0.128; *p* < 0.001), changes in physical environment (β = 0.041; *p* = 0.005), trimester (β = −0.192; *p* = 0.001), and Gestational Diabetes Mellitus (GDM) (β = 0.408; *p* < 0.001) explained 52.1% variance in initiating PA behavior. Emotional transformation (β = 0.197; *p* < 0.001), practice for change (β = 0.083; *p* = 0.001), changes in social environment (β = 0.063; *p* < 0.001), pre-pregnancy exercise habit (β = −0.251; *p* = 0.001), and GDM (β = 0.298; *p* = 0.003) were significantly associated with pregnant women’s intentions to maintain PA behavior and explained 49.1% variance. Conclusions: The constructs of MTM were effective in explaining the intention to initiate and maintain PA behavior among Chinese pregnant women.

## 1. Introduction

It has been proven that maternal and infant health benefits greatly from physical activity (PA) during pregnancy. Being physically active during pregnancy can reduce the risks of adverse pregnancy and birth outcomes such as preeclampsia, Gestational Diabetes Mellitus (GDM), and premature delivery [1]. PA can also affect mental health and prevent postpartum depression [2]. In addition, PA helps control excessive weight gain during pregnancy [3]. Therefore, the appropriate PA level was beneficial to pregnant women’s health on the short-term and the long-term [4].

PA has been shown to be beneficial to pregnant women, but maternal exercise compliance has not been positive in previous studies. About 56% of pregnant women in the United States take exercise, 59% in Norway, and 85% in Canada, yet only about 15% to 28% of pregnant women actually reach the guidelines’ standard [5]. According to Zhang et al. [6], only 11.7% of pregnant women met the guidelines-recommended standard of exercise in Tianjin, China. Even pregnant women who had regular exercise habits before pregnancy reduced the intensity and frequency of PA after pregnancy [7]. 

According to some studies, women who are pregnant reduce their PA for several reasons. For instance, a study among 1535 pregnant women showed that 85% of the women reported an intrapersonal barrier to PA. A total of 2% of the participants reported interpersonal reasons as their main barrier to PA, while for 3% of the participants the neighborhood or an environmental barrier was the main barrier [8]. Besides, research conducted in South Africa has shown that the providers’ lack of awareness of current American College of Obstetricians and Gynecologists (ACOG) recommendations was also a reason for poor exercise compliance [9].

**Figure 1:**
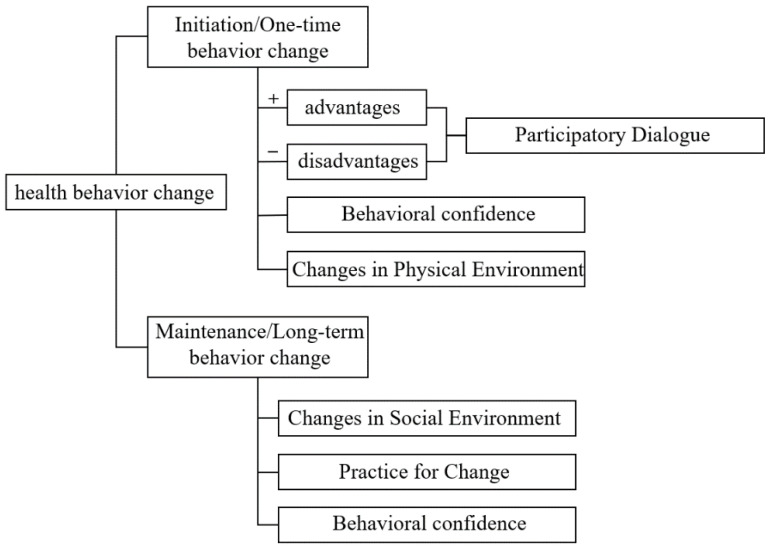
The framework of MTM.

In order to improve compliance with PA during pregnancy, it is indispensable to explain the health behavior change (HBC) from the theoretical level. The interventions in health management has developed over four generations, from knowledge-based to skills-based to a single theory-based, and to the current precise intervention based on multiple theories. The Multi-Theory Model (MTM) is an emerging behavioral–theoretical model that was designed by Manoj Sharma in 2015 [10]. It is a theory of HBC that can be used to understand the factors related to the initiation and maintenance of HBC. Initiation of HBC involves switching from one behavior to another. Maintenance of HBC is to make sure the behavioral change continues over the long term. As the fourth-generation theoretical model, MTM extracts from previous theories’ “optimal variables” and forms a unified, concise theoretical framework, which is a good tool for understanding HBC. Since the advent of MTM, researchers from various countries have verified it in different populations, mainly focusing on several aspects such as exercise, healthy diet, substance addiction management, mental health, and medical compliance [11,12,13,14,15,16,17,18,19,20,21,22,23,24]. Their studies obtained good results and indicated MTM could be used in different populations, however, the effectiveness in pregnant women was still unknown. Therefore, this study aimed to identify the utility of MTM to predict intention to undertake PA behavior in Chinese pregnant women. The framework of MTM is shown in Figure 1.

## 2. Materials and Methods

### 2.1. Study Design and Population

It was a cross-sectional study conducted in a university hospital in Hangzhou, Zhejiang Province, China. Convenience sampling was adopted to recruit the participants. The inclusion criteria for pregnant women were: (1) aged 20 or above; (2) had conceived naturally; (3) had a singleton pregnancy; (4) completed less than 150 min per week of PA. The exclusion criteria were miscarriage; an exercise contraindication such as heart disease, preeclampsia, hypertension, placenta previa, etc.; a history of psychiatric and psychological disorders; visual impairment or comprehension impairment; and participating in another trial before enrolment.

### 2.2. Instrument

#### 2.2.1. General Questionnaire

A self-composed questionnaire was used to obtain the participants’ data comprising place of residence, age, nationality, religion, trimester, educational level, pre-pregnancy body mass index (BMI), pre-pregnancy exercise habit, parity, GDM (the diagnostic standard proposed by the International Association of Diabetes and Pregnancy Study Group (IADPSG)) [25], etc.

#### 2.2.2. Measuring Change in Physical Activity Questionnaire

The Measuring Change in Physical Activity Questionnaire (MCPAQ) was used for the assessment of intentions to undertake PA. It was originally developed in English based on the MTM construct [10]. The 29-item scale included two subscales, the initiation subscale, and the maintenance subscale. It was first developed for college students and was verified in the PA of African–American women. Yang et al. [26] obtained authorization from the original authors of MCPAQ and conducted a cross-cultural adaptation to form a Chinese version of the MCPAQ. It was verified in hypertensive patients and showed good reliability and validity. For the total scale, Cronbach’s alpha = 0.83, and the subscales ranged from 0.63 to 0.92. 

Five items assessed the participatory dialogue-disadvantages. For example, “If you participate in more than 150 min of moderate to vigorous intensity aerobic PA every week you will be tired.” Items ranged from never (=0) to very often (=4). The scores for each question were added together to obtain the total possible score for the disadvantages (ranging from 0 to 20). The score of advantages minus disadvantages was the total score of the participatory dialogue. 

Five items assessed behavioral confidence. For example, “How confident are you about getting 150 min of moderate to intense aerobic activity this week?” The response for each item ranged from not at all sure (=0) to completely sure (=4). The scores for each item were added together to obtain the total possible score for behavioral confidence (ranging from 0 to 20). 

Three items assessed the changes in physical environment. For example, “How sure are you that you will have a place to do 150 min of aerobic exercise every week?” Items ranged from not at all sure (=0) to completely sure (=4). The scores of each item were added to obtain the total possible score of the physical environment (ranging from 0 to 12). 

Emotional transformation was assessed with three items. For example, “How sure are you that you can direct your emotions/feelings towards the goal of aerobic exercise for 150 min a week?” Items ranged from not at all sure (=0) to completely sure (=4). The scores of each item were added together to obtain the total possible score of emotional transformation (ranging from 0 to 12).

Three items assessed practice for change. For example, “How sure are you that you can keep a self-diary to monitor total time of your aerobic physical activity every week?” Items ranged from not at all sure (=0) to completely sure (=4). The scores of each item were added together to obtain the total possible score of practice for change (ranging from 0 to 12). 

Three items assessed changes in the social environment. For example, “How sure are you that you can get the help of a family member to be aerobically physically active for 150 min every week?” Items ranged from not at all sure (=0) to completely sure (=4). The scores of each item were added together to obtain the total possible score of social environment (ranging from 0 to 12).

The final two questions were added to access the initiation construct and maintenance construct. For example, “How likely is it that you will increase your aerobic physical activity to 150 min in the upcoming weeks? “The items ranged from 0 (never) to 4 (very often).

### 2.3. Data Collection

Data collection was conducted in the Obstetric Outpatient clinic of the hospital. The “pen and paper” questionnaires were distributed by two well-trained researchers from March 2022 to June 2022. The researchers explained the purpose of the survey to the participants and guided them on completion of the questionnaires. The survey was voluntary and anonymous. Thirty pregnant women were conveniently selected for retest at intervals of 2 weeks. 

### 2.4. Data Analysis

Data analysis was conducted using IBM SPSS Statistics 25 and IBM AMOS 26. Continuous variables are represented by the mean and standard deviation (SD), and categorical variables are described by percentage frequency. The stepwise multiple regression model was used to model the association between outcome and independent variables. The level of statistical significance was *p* ≤ 0.05. 

#### 2.4.1. Construct Validity

The construct validity of an instrument indicates whether its scores accurately reflect the dimensions of the measured construct. We used maximum likelihood confirmatory factor analysis (CFA) to test the construct of the scale. According to the previous study, we use the following indices and criteria to evaluate the model fit, including χ^2^/df, root mean square error of approximation (RMSEA), comparative fit index (CFI), goodness-of-fit index (GFI), and standardized root mean square residual (SRMR). The model fit would be acceptable if 1 < χ^2^/df < 3, RMSEA and SRMR < 0.08, CFI and CFI > 0.90 [27].

#### 2.4.2. Reliability 

Generally, reliability refers to how stable and consistent the results are measured by the scale. Cronbach’s alpha and test-retest reliability were used to evaluate the consistency of the scale. A Cronbach’s alpha value of 0.70 or higher and test-retest reliability value greater than 0.75 were considered acceptable [28]. 

#### 2.4.3. Floor/Ceiling Effect

When a questionnaire has a maximum score limit and a large number of respondents’ scores are close to this maximum score, this is known as the ceiling effect. The opposite situation is called the floor effect. The floor effects for the scale were calculated by the percentage of the sample size with the lowest score, and the ceiling effects were assessed by the percentage of the respondents with the highest score. Less than 15% of patients achieved the highest or lowest score in entire scale, so it was considered that there were no floor and ceiling effects.

### 2.5. Ethical Consideration

This study was approved by the Ethics Committee of a university hospital in Hangzhou, Zhejiang Province, China (No. 20220666). All participants provided informed consent before their enrolment in the study.

### 2.6. Sample Size

In general, the minimum sample size for factor analysis should have at least 5 times as many as the number of variables [29]. In addition, it is recommended to use two independent samples instead of one to perform EFA and CFA while analyzing the psychometric properties. Therefore, a sample size of at least 145 participants for EFA and CFA was required to estimate the psychometric properties of the 29-item MCPAQ. In order to avoid missing responses, we conveniently recruited 450 participants from 480 eligible pregnant women with a response rate of 93.75%.

## 3. Results

### 3.1. Demographics, and Descriptive Statistics

A total of 406 pregnant women participated. The age ranged from 20 years to 45 years (M = 30.77 years, SD = 4.00 years). Half of them (*n* = 239) were in their second trimester, followed by the third trimester (*n* = 163). Participants who exercised during pregnancy (*n* = 226) were almost the same as those who did not (*n* = 224). A high proportion of the women (*n* = 409) were employed. According to the pre-pregnancy BMI, 71.3% of them had normal pre-gestational BMI (*n* = 321) (Table 1). 

### 3.2. Reliability

In this study, the Cronbach’s alpha value of the MCPAQ was 0.853 for the total scale and ranged from 0.755 to 0.960 for all subscales, respectively (Table 2). To examine test-retest stability, 30 participants’ samples were retested after 2 weeks. The score correlation coefficient of consistency of the scale before and after was 0.909. These results showed that the internal reliability of MCPAQ was good. 

### 3.3. Validity

#### 3.3.1. Exploratory Factor Analysis (EFA)

Sample 1 was used to perform the EFA. For the initiation model, the result of EFA showed that the Kaiser–Meyer–Olkin value = 0.900 and the significance of Bartlett’s test of sphericity was *p* < 0.001 (χ^2^ = 3232.391, df = 153), demonstrating a four-factor structure. Factor 1 included five items and was labeled “participatory dialogue-advantage”. Factor 2 contained five items, which were labeled “Behavioral Confidence”. Factor 3 contained five items and was labeled “Participatory Dialogue-disadvantage”. Factor 4 contained three items, labeled “Changes in Physical Environment”. The contribution rate of the total cumulative variance was 77.438%, and the four common factors accounted for 23.256%, 22.155%, 19.179%, and 12.849% of the total variance, respectively (Table 3).

For the maintenance model, the result showed Kaiser–Meyer–Olkin value = 0.822, and the significance of Bartlett’s test of sphericity was *p* < 0.001 (χ^2^ = 1268.075, df = 36), demonstrating a three-factor structure. These results indicated an adequate sample size for the factor analysis. Factor 1 included three items and was labeled “Emotional Transformation”. Factor 2 contained three items, which was labeled “Change for Practice”. Factor 3 contained five items and was labeled “Changes in Social Environment”. The contribution rate of the total cumulative variance was 77.589%, and the four common factors accounted for 29.379%, 25.684%, and 22.525% of the total variance, respectively (Table 4).

#### 3.3.2. Confirmatory Factor Analysis (CFA)

**Figure 2:**
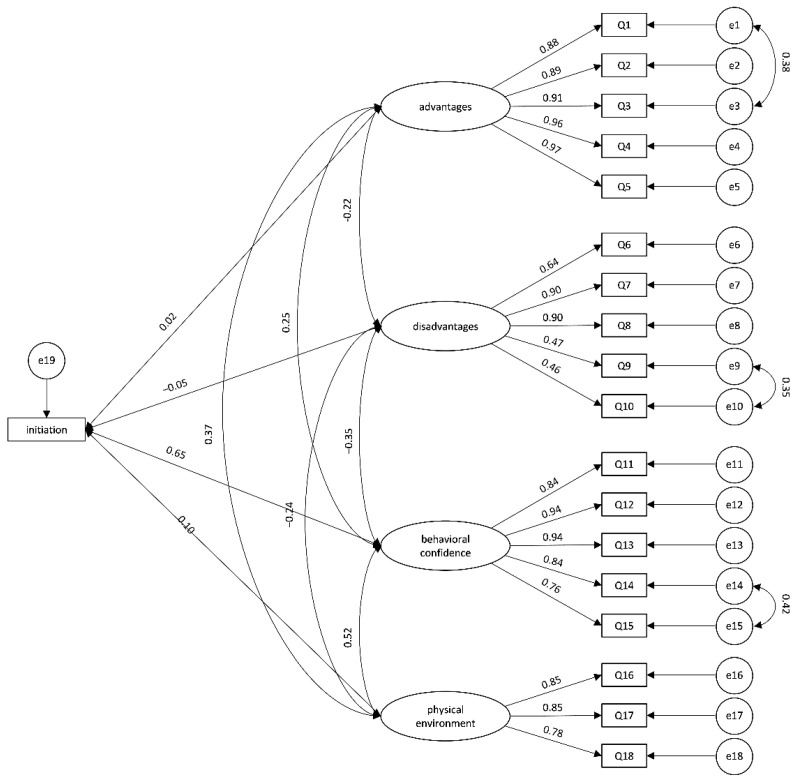
Structural equation model for initiation model.

Sample 2 was used for CFA, using Maximum Likelihood Estimates to verify the construct validity of the factor model. For the initiation model, the initial model fitting was not ideal. Covariations between items 1 and 3, items 9 and 10, and items 14 and 15 were added according to modification indices to re-examined fit indices. The results showed the four-factor model provided a good fit across fit indices [χ^2^ = 206.123, df = 140, *p* < 0.001, χ^2^/df = 1.472, RMSEA = 0.046, SRMR = 0.0432, GFI = 0.913, CFI = 0.982] (Figure 2).

**Figure 3:**
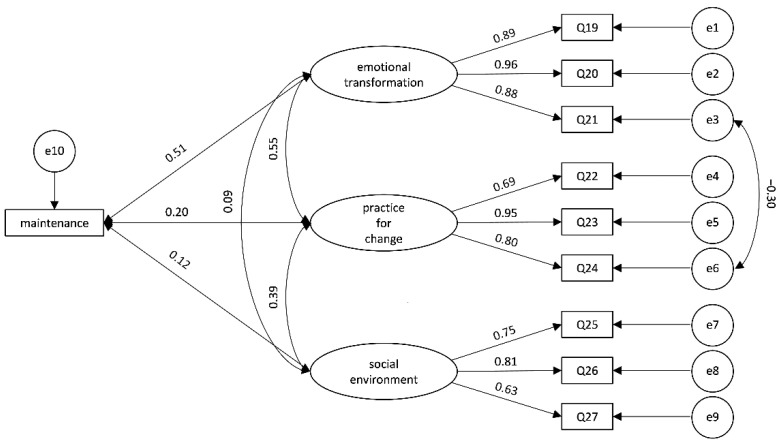
Structural equation model for maintenance model.

For the maintenance model, we paired items 1 and 3, items 9 and 10, and items 14 and 15, showing improvement in all indices by examining the model fitting statistics, and achieving a better acceptable fitting [χ^2^ = 49.742, df = 29, *p* < 0.001, χ^2^/df = 1.715, RMSEA = 0.057, SRMR = 0.0432, GFI = 0.958, CFI 0.985] (Figure 3).

### 3.4. Stepwise Multiple Regression

For the initiation model, the results showed that participatory dialogue, behavioral confidence, changes in physical environment, gestation age, and GDM (F (5, 434) = 94.551, *p* < 0.001) accounted for 52.1% of the variance of initiating PA. The detailed results are presented in Table 5.

Dependent variable: intention of initiating PA behavior; Significant at a *p*-value less than 0.05.

For the maintenance model, the result showed that emotional transformation, practice for change, changes in social environment, pre-pregnancy exercise habits, and GDM (F (5, 435) = 83.608, *p* < 0.001) accounted for 49.1% of the variance of initiating PA. Table 6 presents the results of stepwise multiple regression analysis for initiation model. 

Dependent variable: intention of maintaining PA behavior; Significant at a *p*-value less than 0.05.

### 3.5. Ceiling/Floor Effect

Both the ceiling effect and floor effect were 0%.

## 4. Discussion

In this study, we used MTM as a theoretical framework to predict PA behavior in Chinese pregnant women. MCPAQ was a questionnaire developed based on MTM. We found MTM fitted the data of this study well and the reliability and validity of MCPAQ were acceptable, without a floor/ceiling effect. The intention of initiating and maintaining PA behavior in this study was at a medium to a high level and was higher than the intention in other parts of the population [10,30]. The model included two parts: initiation and maintenance of behavioral change. For the initiation model, gestation age, GDM, and all the constructs in MTM were considered significant which can explain 52.1% of the variance in initial intention. For the maintenance model, pre-pregnancy exercise habits, GDM, and all the constructs proposed in the maintenance model were significant and accounted for 49.1% of the variance of intention to maintain. In general, MTM accounted for a moderate amount of variance, which indicates that it is a useful theory to explain PA behavior in Chinese pregnant women. The percentage variance predicted by both models is similar to what has been observed in earlier studies conducted with the theory of planned behavior, social cognitive theory, and the health belief model [31,32,33].

In our study, we found that the gestation age was significantly related to the intention of PA behavior, and PA intention decreased gradually with the increase in gestation age. Some studies supported this view [34,35,36]. As the gestational week increases, weight gain and changes in cardiopulmonary function may result in a lower intention of PA behavior [37]. Guidelines suggest that pregnant women with no exercise contraindications should be encouraged to maintain PA during the whole pregnancy, and the type and intensity of exercise can be appropriately adjusted according to the gestational age and physical condition [38,39]. Consequently, to increase PA during pregnancy, researchers should focus on the second and third trimesters and take measures to improve pregnant women’s PA intention in these two stages.

GDM was significant in predicting behavioral intention in both models. Pregnant women diagnosed with GDM had a higher intention to initiate PA behavior than those who were not diagnosed. Lifestyle interventions, including diet and PA intervention, are preferred for the management of GDM. Once GDM is diagnosed, pregnant women would receive health education on lifestyle changes; therefore, they have higher exercise intentions for the health of the women and fetuses [40].

Pre-pregnant exercise habits were a significant predictor of intent to initiate PA behavior. Consistent with previous studies, pregnant women with pre-pregnancy exercise habits tended to be more active during their pregnancy than women without exercise habits [6,41]. Thus, it is necessary to encourage women of reproductive age, particularly those planning to become pregnant, to be physically active before getting pregnant. Regular exercise habits bring a long-term impact for people. In the future, we should strengthen national health education, especially incorporating exercise into prenatal education.

In the current study, the three constructs proposed by MTM in the initiation model were found to be crucial. The dialogue was initiated by health educators and involved mutual communication about the advantages and disadvantages of behavioral change [10]. This result was supported by two studies based on the Transtheoretical model and the Health Belief Model [42,43]. The advantages of PA during pregnancy should be communicated more by medical workers to pregnant women. The construct of behavioral confidence is similar to perceived behavioral control and self-efficacy [10]. Consistent with the studies by Lee [44] and Gaston [45], we found that PA in pregnant women was related to increased behavioral confidence in the health benefits. Further research is needed on increasing the confidence in PA behavior during pregnancy. For changes in the physical environment, changing the availability, usability, accessibility, convenience, and readiness of related resources in the physical environment have been proven as predictors of improving PA intentions [30]. Government organizations should take a coordinated approach to the construction of national fitness venues and facilities, build a higher level of public service systems for national fitness, and improve the accessibility of fitness facilities.

All constructs in the maintenance model were also significant variables. Emotional transformation, derived from the self-motivation of Emotional Intelligence Theory (EIT), refers to overcoming self-doubt, inertia, and impulse, focusing one’s feelings and emotions on the changes in healthy behaviors. It is crucial to help pregnant women record their daily PA achievements by keeping pregnancy diaries and assisting them to strengthen self-supervision. Practice for change emphasizes reflective behavior, including the continuous and prudent consideration of behavioral change, combined with a continuous correction to remove ineffective strategies and solve obstacles. These results have been proven to be effective in other people’s PA behaviors [10,30]. Changes in social environment refer to the establishment of social support in the environment. Strategies to strengthen the support of family numbers, friends, and health educators to be physically active during pregnancy are required [6,46].

### Strengths and Limitations

To the best of our knowledge, this study is the first to understand and explain the intention of PA behavior in pregnant women based on MTM, and also the first to test the applicability of MTM in China. The results verify the good cultural applicability of MTM; it retains positive effects in different cultural environments. Although the predictors were similar to those reported in other populations, our study confirms these predictors among Chinese pregnant women, which means that the interventions that have taken effect in PA promotion among other populations can be applied to pregnant women. The information provided by this study can be used by government organizations and health-care providers to promote PA during pregnancy and support pregnant women to pay more attention to PA. 

This study also had some limitations that need to be discussed. First of all, the participants in this study were confined to one hospital in Hangzhou, Zhejiang Province, China, with a small sample size. In the future, participants from different regions and a larger sample size should be adopted. Moreover, the study relied on subjective self-reporting rather than objective measurements of behavior, which can be biased or exaggerated. Meanwhile, in a cross-sectional design study, the independent and dependent variables were collected simultaneously, which cannot determine the temporality of the association.

## 5. Conclusions

In summary, MTM could be suitably applied to determine the predictors of intention PA during pregnancy. The key predictors for the intention of the PA behavior initiation included participatory dialogue, behavioral confidence, changes in physical environment, gestation age, and GDM. Emotional transformation, practice for change, changes in social environment, pre-pregnancy exercise, and GDM were key predictors of PA behavior maintenance. In order to promote PA behavior, future interventions should take into account these modifiable factors in HBC interventions to improve the PA of Chinese pregnant women.

## Figures and Tables

**Table 1:** Characteristics of participants (*n* = 450).

Variables	*n*	%
Age (years)		
<35	375	83.3
≥35	75	16.7
Education		
Junior high school or less	19	4.2
Senior high school/Specialized Secondary School	36	8.0
College/University	339	75.3
Graduate school	56	12.4
Nationality		
Han	441	98.0
National minority	9	2.0
Religion		
Atheist	372	82.7
Christianity	11	2.4
Buddhism	37	8.2
Other	30	6.7
Residence		
Urban	373	82.9
Rural	77	17.1
Working Status		
Employed	410	91.1
Student	1	0.2
Unemployed	40	8.9
Monthly family income per capita (CNY)		
≤4000	12	2.7
4000–8000	115	25.6
8000–12,000	156	34.7
≥12,000	167	37.1
Pre-pregnancy BMI (kg/m^2^)		
Underweight (<18.5)	70	15.6
Normal (18.5–23.9)	321	71.3
Overweight and Obese (≥24.0)	59	13.1
Pre-pregnancy exercise habit		
Yes	226	50.2
No	224	49.8
Parity		
1	327	72.7
>1	123	27.3
Medical insurance		
Yes	411	91.3
No	39	8.7
Gestation age		
First trimester (≤13)	48	11.8
Second trimester (14–27)	239	53.1
Third trimester (≥28)	163	36.2
GDM ^1^		
Yes	80	17.8
No	370	82.2

^1^ GDM: Gestational Diabetes Mellitus.

**Table 2:** Multi-Theory Model Constructs (*n* = 450).

Constructs	Possible Range	Observed Range	Mean (SD)	Cronbach’s Alpha
Initiation	0 to 4	0 to 4	2.30(1.08)	N.A. ^1^
Participatory Dialogue: Advantages	0 to 20	0 to 20	16.56(4.01)	0.960
Participatory Dialogue: Disadvantages	0 to 20	0 to 20	6.96(4.01)	0.850
Participatory Dialogue: Advantages—Disadvantages score	−20 to 20	−20 to 20	9.60(6.47)	N.A.
Behavioral confidence	0 to 20	0 to 20	11.31(4.74)	0.938
Change in Physical Environment	0 to 12	0 to 12	7.22(2.91)	0.861
All constructs of Initiation Model	N.A.	N.A.	N.A.	0.766
Maintenance	0 to 4	0 to 4	2.24(1.09)	N.A.
Emotional Transformation	0 to 12	0 to 12	7.96(2.35)	0.937
Practice for Change	0 to 12	0 to 12	4.00(2.50)	0.834
Change in Social Environment	0 to 12	0 to 12	7.07(2.70)	0.755
All Constructs of Sustenance Model	N.A.	N.A.	N.A.	0.863
Entire Scale	N.A.	N.A.	N.A.	0.857

^1^ N.A.: Not Applicable.

**Table 3:** Results of the EFA and factor loading for the MCPAQ-INIT (*n* = 225).

Item	Factor Loading			
	Factor 1	Factor 2	Factor 3	Factor 4
Q4	0.911			
Q5	0.901			
Q3	0.881			
Q1	0.874			
Q2	0.860			
Q13		0.856		
Q12		0.840		
Q11		0.837		
Q14		0.837		
Q15		0.826		
Q7			0.843	
Q8			0.839	
Q6			0.809	
Q10			0.770	
Q9			0.718	
Q18				0.819
Q17				0.816
Q16				0.787

**Table 4:** Results of the EFA and factor loading for the MCPAQ-MAINT (*n* = 225).

Item	Factor Loading		
	Factor 1	Factor 2	Factor 3
Q19	0.881		
Q20	0.881		
Q21	0.881		
Q23		0.836	
Q24		0.817	
Q22		0.801	
Q26			0.870
Q27			0.776
Q25			0.677

**Table 5:** Multiple Regression Model of intention to initiate PA Behavior (*n* = 450).

Variables	B	SE_B_	β	95% CI for B	*p*-Value
Participatory dialogue	0.030	0.009	0.118	0.012, 0.049	0.002
Behavioral confidence	0.128	0.009	0.560	0.109, 0.146	<0.001
Changes in Physical Environment	0.041	0.015	0.111	0.013, 0.070	0.005
Gestation age	−0.192	0.057	−0.112	−0.304, −0.080	0.001
GDM	0.408	0.096	0.142	0.218.0.598	<0.001

F (5, 434) = 94.551, *p* < 0.001, R^2^ (Adjusted R^2^) = 52.1% (51.6%).

**Table 6:** Multiple Regression Model of intention to maintain PA Behavior (*n* = 450).

Variables	B	SE_B_	β	95% CI for B	*p*-Value
Emotional Transformation	0.197	0.021	0.424	0.156, 0.238	<0.001
Practice for Change	0.083	0.018	0.188	0.047, 0.118	<0.001
Changes in Social Environment	0.063	0.016	0.155	0.031, 0.094	<0.001
Pre-pregnancy exercise habit	−0.251	0.074	−0.121	−0.396, −0.106	0.001
GDM	0.298	0.100	0.103	0.102, 0.495	0.003

F (5, 434) =83.608, *p* < 0.001, R^2^ (Adjusted R^2^) = 49.1% (48.5%).

## Data Availability

The data are not publicly available due to privacy and ethical restrictions.
